# Species Identification and Spatial Diversity Patterns of the Giant Panda National Park (GPNP) in Chengdu, Sichuan, China

**DOI:** 10.1002/ece3.73180

**Published:** 2026-02-27

**Authors:** Qianqian Wang, Chi Xu, Yubo Gou, Mingchun Zhang, Ke He, Qiujie Li, Jingjing Shuai, Chun Yin, Zhaowen Wang, Zhisong Yang, Biao Yang

**Affiliations:** ^1^ Key Laboratory of State Forestry and Grassland Administration for the Giant Panda China Conservation and Research Center for the Giant Panda Chengdu China; ^2^ Sichuan Provincial Research Institute of Giant Panda Sciences Chengdu China; ^3^ Chengdu Administration Bureau of Giant Panda National Park Chengdu China; ^4^ College of Life Sciences China West Normal University Nanchong China; ^5^ Society of Entrepreneurs and Ecology (SEE) Foundation Beijing China

**Keywords:** biodiversity conservation, Chengdu, Giant Panda National Park, giant pandas and sympatric species, habitat connectivity, species turnover

## Abstract

Elucidating the spatial patterns of biodiversity and their driving mechanisms is crucial for predicting the impacts of environmental changes and informing conservation and management efforts. This study investigated the spatial distribution patterns of species in the Chengdu section of the Giant Panda National Park (GPNP), located in the western part of Sichuan Province, China, which provides a wide range of suitable habitats for diverse species and biotic communities. Species trace points data derived from the 4th National Survey on Giant Pandas were used to analyze the diversity and composition of mammals and gallinaceous birds among the five regions. The results indicated that the central regions comprising Dujiangyan Shi (DJYS), Chongzhou Shi (CZS), and Dayi Xian (DYX) exhibited higher α‐diversity, whereas Qionglai Shi (QLS) and Pengzhou Shi (PZS) presented lower values across most indices, a pattern that was consistent with expectations of the mid‐domain effect. However, Pielou's evenness index showed no significant differences among regions. β‐diversity analysis revealed that species turnover was the dominant factor contributing to faunal heterogeneity among regions, with nestedness playing a relatively minor role. These findings highlight the necessity for coordinated conservation among the five regions within the Chengdu section of the GPNP, with a particular focus on improving habitat connectivity and establishing species dispersal corridors to maintain biodiversity. The study provides valuable insights for the development of targeted conservation strategies and the establishment of a long‐term biodiversity monitoring system, which will enhance the ecological sustainability of the GPNP and contribute to the protection of isolated giant panda populations and their sympatric species.

## Graphical abstract

This study investigates the spatial distribution patterns of species in the Chengdu section of the Giant Panda National Park (GPNP) in China, focusing on mammals and gallinaceous birds. The results show that central regions like Dujiangyan Shi and Chongzhou Shi exhibit higher species diversity, while peripheral regions like Pengzhou Shi and Qionglai Shi have lower diversity. The findings highlight the importance of enhancing habitat connectivity and establishing ecological corridors to support biodiversity conservation in the GPNP.

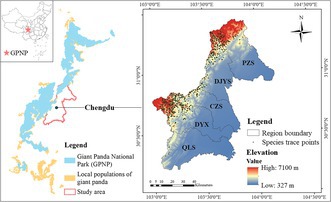

## 1 Introduction

Biodiversity is a critical component of ecosystems, and understanding its spatial distribution and the factors that influence it is essential for developing effective conservation and management strategies (Gaston 2000; Jetz et al. 2012; Mahmoodzadeh et al. 2022; Parmesan 2006). Mountain ecosystems, often characterized by high species richness and endemism, have emerged as model regions for studying the complex interplay among biodiversity, environmental gradients, and ecosystem functioning (Gaston et al. 2008; McCain 2005; Ronchi and Brambilla 2025). In the context of accelerating global climate change, these ecosystems are increasingly threatened by habitat fragmentation due to natural processes and human activities, such as deforestation and infrastructure development, and their high sensitivity to these changes makes them important areas for biodiversity research and conservation (Cardinale et al. 2012; Hingnekar and Dhadse 2025; Myers et al. 2000; Pimm et al. 2014).

National parks and nature reserves serve as vital sanctuaries for wildlife conservation, offering protected habitats that support biodiversity and species survival, while also enabling continuous monitoring and research (Cao et al. 2025; Parsons 2004). The Giant Panda National Park (GPNP) in China serves as a prime example of a mountainous region with significant conservation importance, exemplified by its role in maintaining ecosystem integrity and protecting critical habitats since the establishment of China's first group of national parks in 2021 (Yang et al. 2023). The giant panda ( *Ailuropoda melanoleuca* ), a flagship species for conservation, epitomizes the broader efforts to safeguard biodiversity (Jiangzuo et al. 2024; Rong et al. 2019). Despite notable conservation milestones, such as the reclassification of the giant panda from Endangered (EN) to Vulnerable (VU) on the IUCN Red List (IUCN 2025), its population remains highly fragmented into numerous local populations (Huang et al. 2023). This fragmentation presents significant challenges for genetic connectivity and long‐term population viability. Therefore, elucidating the spatial diversity patterns of giant panda populations and their sympatric species, along with the characteristics of their associated habitats, is crucial for guiding targeted conservation interventions and promoting regional sustainable development (Wang et al. 2024).

Species richness and evenness, which are fundamental indicators of biodiversity and represent α‐diversity, are commonly used to predict species distribution patterns (Gaston and Blackburn 2000; Zhang et al. 2014). Meanwhile, β‐diversity, which reflects the differences in species composition among communities or ecosystems, provides additional insights into the spatial variation of biodiversity (Pinto et al. 2020; Zhao et al. 2022). An increasing number of studies have utilized α‐ and β‐diversity to monitor biodiversity trends, explore their patterns and mechanisms across spatial, temporal, and environmental gradients, and assess the impacts of human activities or global changes on biodiversity, as well as the effectiveness of conservation priority areas (Enkhtur et al. 2021; Podani and Schmera 2011). β‐diversity decomposes into turnover and nestedness, with the former elucidating species replacement along spatial or environmental gradients and the latter reflecting ordered species loss across the species richness gradient, thereby providing essential insights into the drivers of biodiversity patterns and informing effective conservation strategies (Chen et al. 2024; Socolar et al. 2016; Si et al. 2017). For example, studies have shown that high β‐diversity in bird metacommunities in China's mountainous regions is primarily driven by spatial factors, with local connectivity being a significant factor affecting bird distribution (Li et al. 2022).

The formation and distribution patterns of species diversity are the result of the interplay between local environmental conditions and larger‐scale regional processes (Babu 2023; Maurer et al. 2013; Rangel and Diniz‐Filho 2005). Previous studies have highlighted the importance of habitat heterogeneity, species attributes, and environmental gradients in shaping the spatial distribution of species (Du et al. 2017; Hawkins et al. 2003; Heaney 2001). While numerous studies have explored the large‐scale distribution patterns of species diversity, research focusing on small‐scale regional patterns remains relatively limited (Beaugrand 2023; Coelho et al. 2023; Rosindell and Cornell 2007). However, investigations at smaller scales such as local or patch levels are particularly crucial as they not only reveal the intricate complexity of ecosystems but also help elucidate the underlying mechanisms of species coexistence or replacement within limited spaces.

The Chengdu section of the GPNP serves as a crucial habitat for local giant panda populations and their sympatric species, as well as for the protection of associated ecosystems. This area also holds significant value for cultural landscapes and associated natural heritage values, acting as a vital corridor for wildlife movement and habitat connectivity. Integrating data from the 4th National Survey on Giant Pandas across five administratively distinct regions, this study first quantifies the spatial diversity patterns of mammals and gallinaceous birds. Specifically, (i) α‐diversity is compared among the five regions, (ii) β‐diversity is partitioned into turnover and nestedness components, and (iii) the resulting patterns are translated into conservation implications for regional connectivity and corridor design. These outcomes will inform long‐term biodiversity monitoring protocols and adaptive management of endangered vertebrate resources throughout the GPNP.

## 2 Materials and Methods

### 2.1 Study Area

**FIGURE 1:**
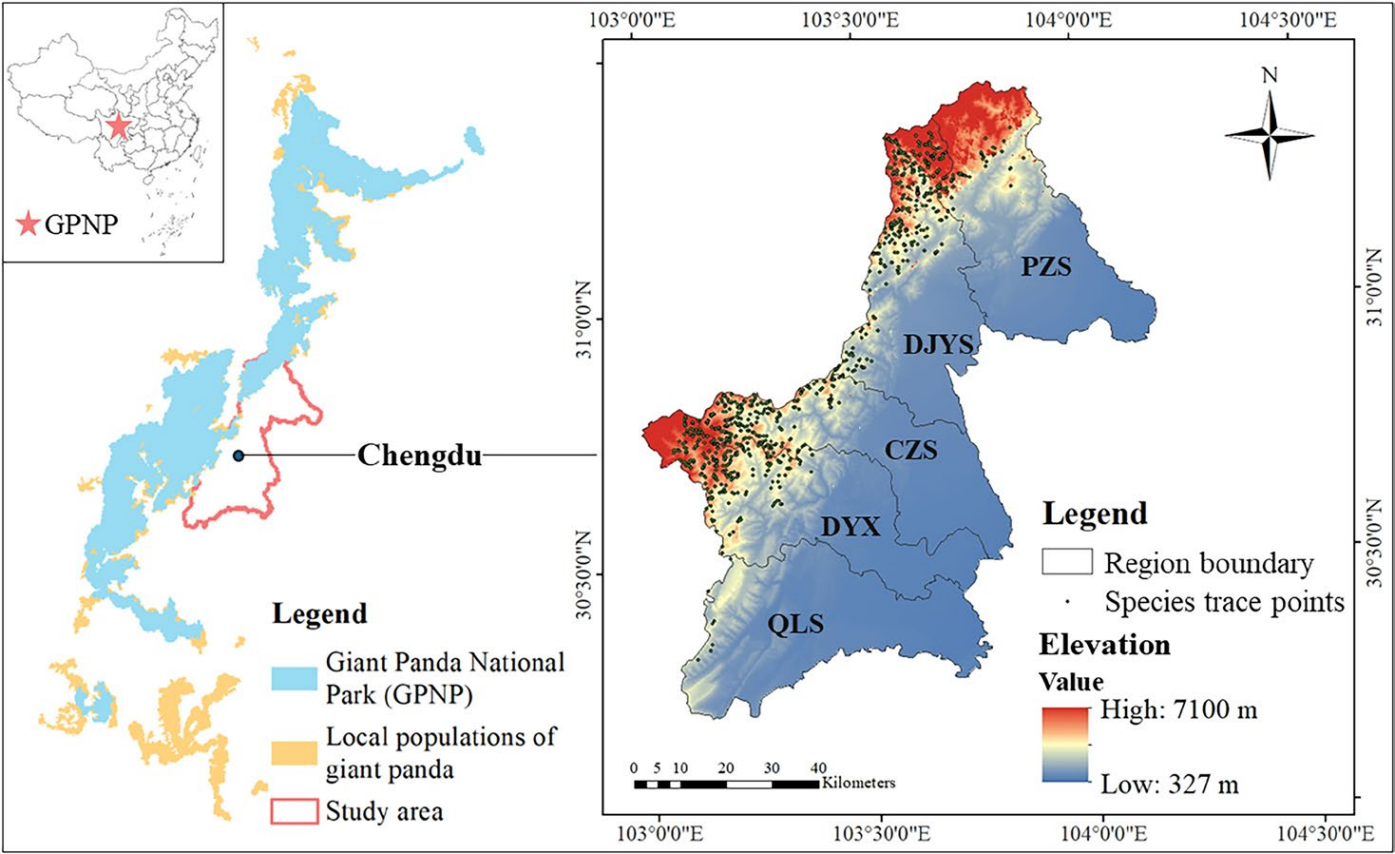
Location and elevation map of the study area within the GPNP in Chengdu, Sichuan, China. The digital elevation model (DEM) data were sourced from the ASTER Global Digital Elevation Model Version 3 (https://www.earthdata.nasa.gov/data/catalog/lpcloud‐astgtm‐003), which has a spatial resolution of 1 arc‐second.

This research focuses on the Chengdu section of the Giant Panda National Park (GPNP), located in the western part of Sichuan Province, China (Figure 1). The elevation within this area ranges from 327 to 7100 m, with the Chengdu section of the GPNP primarily located in the western mountainous areas at higher elevations. These areas are characterized by complex terrain and diverse climatic conditions, which contribute to the formation of suitable habitats for a variety of wildlife. The Chengdu section of the GPNP serves as a critical ecological corridor, particularly connecting the Min and Qionglai mountain ranges, and plays a vital role in safeguarding isolated giant panda populations. Administratively, Chengdu is divided into five regions including county‐level cities and counties, all of which are of comparable size reflecting historical administrative divisions and natural geographic boundaries. These regions are Pengzhou Shi (PZS), Dujiangyan Shi (DJYS), Chongzhou Shi (CZS), Dayi Xian (DYX), and Qionglai Shi (QLS), each with distinct management resources including ranger stations, patrol zones, and conservation budgets, allowing biodiversity patterns across these boundaries to be related to management effectiveness and resource allocation.

### 2.2 Data Sources

The data on trace points for giant pandas and their sympatric species were obtained from the 4th National Survey on Giant Pandas, collected between 2011 and 2014 (Sichuan Forestry Department 2015). This dataset offers comprehensive information on wildlife distribution that represents the most recent Chinese government‐led census across the GPNP, as the 5th National Survey on Giant Pandas has not yet been conducted (China National Forestry and Grassland Administration 2021). The 4th National Survey on Giant Pandas described field investigation methods conducted by experienced and trained conservation professionals, with standardized manuals highlighting diagnostic features of mammals and gallinaceous birds to ensure reliable identification of different species. More than two thousand survey routes were established, widely and evenly covering all potential giant panda habitats, thereby ensuring comprehensive coverage of both giant panda habitats and sympatric wildlife species. Surveyors utilized advanced handheld PDA devices to document routes, locations, times, photographs, and additional data in real time, promptly uploaded these records to the database, and collected samples for noninvasive DNA analysis, thereby improving field survey and species identification accuracy and efficiency.

The wide‐ranging survey employed a combination of field investigation methods, which required traversing various habitats of giant pandas and sympatric species to record both direct sightings of live animals and indirect signs such as footprints, hair, feces, bite marks, and feeding traces (Fu et al. 2022; Luo et al. 2024; Xu et al. 2022; Yang et al. 2020). According to the results of this survey, a total of 959 trace points were recorded within the Chengdu section of the GPNP, including 213 wild giant panda trace points and 746 sympatric species trace points, representing 28 species distributed across 5 orders and 15 families. To account for unavoidably nonuniform sampling effort across the study area, trace points were collapsed to binary presence per appropriately sized grid cell, with multiple traces of the same species within one grid cell consolidated to a single presence/absence record to avoid pseudo‐replication.

### 2.3 Statistical Analysis

The study area was divided into 5 km × 5 km grid cells using ArcGIS 10.8 software, a scale that balances the focal taxa's movement ecology with detectability constraints, and the five regions within the GPNP in Chengdu were divided into grid cells of varying numbers, with those having an area less than one grid cell being recorded as a single unit. To assess the diversity and dissimilarity in species composition among the five regions within the GPNP in Chengdu, α‐diversity and β‐diversity were calculated using the methods described by Wang et al. (2024). Specifically, α‐diversity was assessed using Chao1 and ACE indices to estimate species richness, Pielou's evenness index to quantify species evenness, and Shannon‐Wiener and Simpson's indices to measure the combined contribution of richness and evenness (Shannon 1948; Simpson 1949; Spellerberg and Fedor 2003). Meanwhile, the Jaccard similarity index (βjac) and the Sørensen similarity index (βsor) were employed to measure β‐diversity, with turnover/replacement (βjtu/βsim) and nestedness/richness difference (βjne/βnes) derived respectively from Jaccard and Sørensen decompositions. Pairwise dissimilarities were calculated between every pair of the five regions to quantify the relative contributions of the decomposed components to the total dissimilarity.

The statistical analyses were also performed in accordance with the approaches outlined by Wang et al. (2024). All statistical analyses and visualizations were conducted using R (version 4.3.3). Diversity analyses were performed using the “vegan” and “betapart” packages (Baselga and Orme 2012; Oksanen et al. 2019). Visualizations were generated using the “VennDiagram,” “reshape2,” and “ggtern” packages (Chen and Boutros 2011; Hamilton and Ferry 2018; Wickham 2007). The Kruskal–Wallis test was employed to evaluate overall differences among the five regions. When significant differences were identified, Dunn's test was subsequently conducted as a post hoc analysis to determine specific pairwise differences among the regions. In the absence of significant results from the Kruskal–Wallis test, additional pairwise comparisons were deemed unnecessary (Lee et al. 2025). Statistical significance was defined with *p* < 0.05 indicating significant differences and *p* < 0.01 indicating highly significant differences (Bates et al. 2020).

## 3 Results

### 3.1 Species Taxonomic Identification and Composition Differentiation of the Five Regions Within the GPNP

A comprehensive species inventory that included mammals and gallinaceous birds from five orders and 15 families revealed a total of 28 species (Table S1). The Primate order was represented by three species, the Carnivora order by nine species, whereas the Artiodactyla order exhibited the highest species richness with 10 species recorded. According to the IUCN Red List, three species, Golden snub‐nosed monkey ( *Rhinopithecus roxellana* ), Forest musk deer ( *Moschus berezovskii* ), and Red panda (*Ailurus styani*), are classified as Endangered (EN). Additionally, eight species, including Sambar ( *Rusa unicolor* ), Chinese serow ( *Capricornis milneedwardsii* ), Takin (*Budorcas tibetana*), Chinese goral ( *Naemorhedus griseus* ), Giant panda ( *Ailuropoda melanoleuca* ), Asiatic black bear ( *Ursus thibetanus* ), Hog badger ( *Arctonyx collaris* ), and Chinese monal pheasant ( *Lophophorus lhuysii* ), are listed as Vulnerable (VU). Furthermore, five species are categorized as first‐class and 13 as second‐class under China's National Key Protected Wildlife List.

**FIGURE 2:**
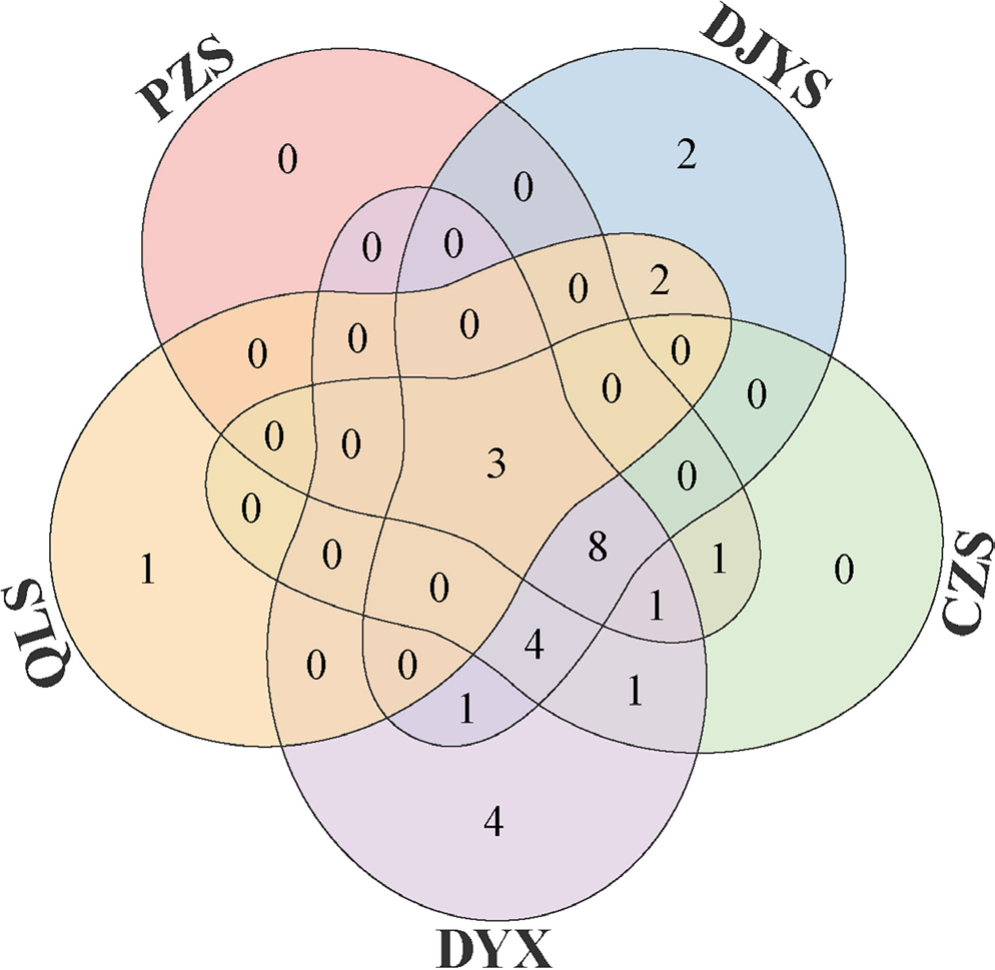
The overlap of species richness among the five regions within the GPNP displayed by the Venn diagram. Region abbreviations are as follows: PZS, Pengzhou Shi; DJYS, Dujiangyan Shi; CZS, Chongzhou Shi; DYX, Dayi Xian; and QLS, Qionglai Shi.

A Venn diagram was employed to examine the core and differential species distribution among the five regions of the GPNP in Chengdu (Figure 2). This graphical representation identified three species that are commonly found among all the five regions. The region of DYX supported the highest species richness, with a total of 22 species recorded. DJYS ranked second with 20 species, while CZS, PZS, and QLS had 18, 13, and 6 species, respectively. In terms of endemic species, DYX was unique in harboring 4 distinct species, followed by DJYS with 2, and QLS with 1. Notably, PZS and CZS lacked any species that were exclusive to these regions, with the species composition of PZS entirely encompassed within that of CZS. From the spatial arrangement of regions, the central cluster of DJYS, CZS, and DYX shares 15 species (3 + 8 + 4). Moreover, these three central regions harbor 4 species that are absent from both peripheral regions PZS and QLS, demonstrating higher overlap toward the geographic core of the Chengdu section.

### 3.2 The α‐Diversity of the Five Regions Within the GPNP

**FIGURE 3:**
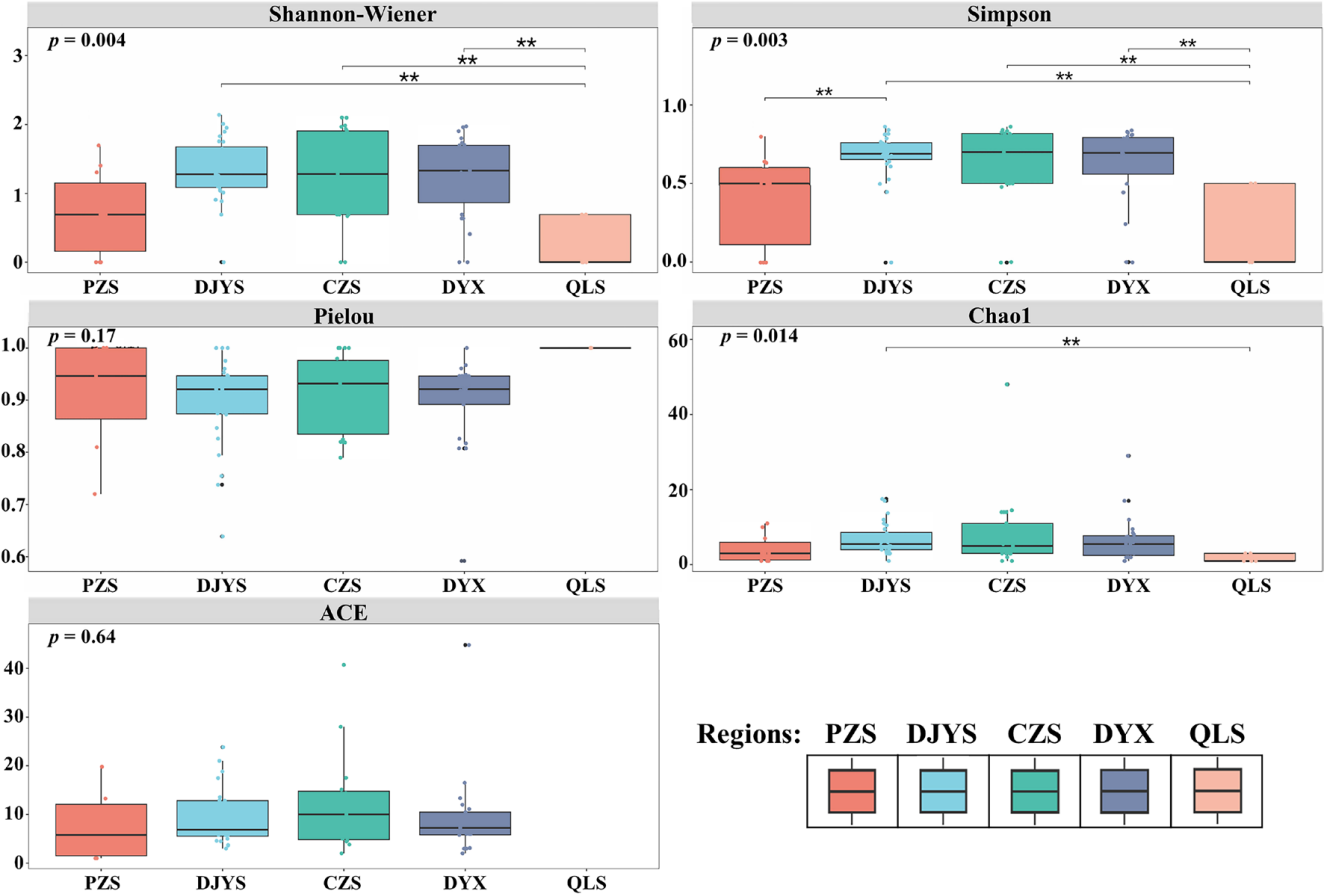
Box plots of α‐diversity indices of the five regions within the GPNP in Chengdu. Each box plot displays the median, interquartile range, and range of the data, with individual grid cells represented by dots. Asterisks indicate statistical significance from pairwise comparisons using Dunn's test: *p < 0.05, **p < 0.01. Note that due to extreme data sparsity, the standard calculation method for the ACE index was not applicable in QLS.

The α‐diversity analysis of the five regions within the GPNP in Chengdu revealed distinct patterns of species diversity. The Shannon‐Wiener and Simpson indices indicated that the region of DJYS had the highest species diversity, followed closely by DYX, while PZS and QLS exhibited lower levels of species diversity. The Chao1 and ACE indices indicated that DYX had the highest estimated species richness. The Pielou index, which measures species evenness, showed the highest value in QLS. Notably, QLS displayed the lowest levels across the Shannon‐Wiener and Chao1 indices, whereas PZS showed the lowest values in Simpson and ACE indices (Table S2). Meanwhile, box plots were utilized to visualize the range, average, and significance of species diversity, evenness, and richness among the different regions (Figure 3). According to the Shannon‐Wiener and Simpson indices, significant differences were observed among regions (Kruskal–Wallis: *p* < 0.01), with CZS, DJYS and DYX exhibiting the highest species diversity and differing significantly from QLS (Dunn: *p* < 0.01). The Chao1 and ACE indices further corroborated the rich species community in CZS and DJYS, with DJYS and QLS showing statistically significant differences in Chao1 (Dunn: *p* < 0.01). However, Pielou's evenness index did not reveal statistically significant differences among the five regions (Kruskal–Wallis: *p* = 0.17). Overall, the central region exhibited significantly higher α‐diversity, while the peripheral regions had lower diversity levels.

### 3.3 The β‐Diversity of the Five Regions Within the GPNP

**FIGURE 4:**
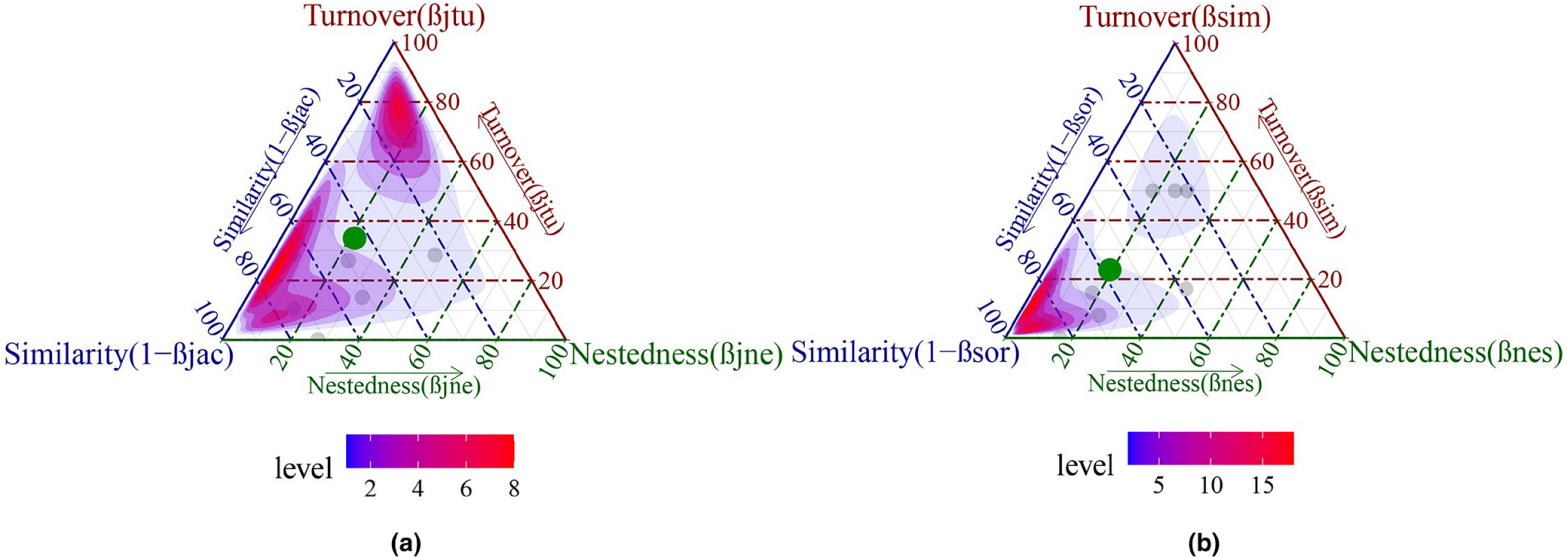
Ternary diagram illustrating the components of β‐diversity for (a) Jaccard (βjac) and (b) Sørensen (βsor) indices. Each point's location within the triangle is defined by the relative proportions of similarity (1−βjac/βsor), turnover (βjtu/βsim), and nestedness (βjne/βnes), with the sum of these proportions equaling 100%. Proximity to a vertex indicates greater dominance of that component in determining β‐diversity. The green dots represent the average values among the five regions. The color gradient represents the density of pairwise comparisons, with blue indicating lower levels and red indicating higher levels.

The components of β‐diversity decomposition of the five regions within the GPNP in Chengdu were assessed using species presence‐absence data, underscoring the predominance of species turnover in contributing to β‐diversity (Table S3; Figure 4). The Jaccard dissimilarity index, which indicated a total dissimilarity value of 0.70175, showed species turnover contributing 64.35% and nestedness 35.65%. Despite variations observed with different β‐diversity measures, the Sørensen dissimilarity index indicated a lower turnover ratio, with species turnover accounting for 53.96% and nestedness for 46.04% of β‐diversity. Nonetheless, the highest pairwise turnover values occurred between QLS and each of the PZS, CZS, and DYX regions (Tables S4 and S5). These findings indicate that spatial species turnover is the primary driver of β‐diversity variation among these regions, whereas nestedness contributes less significantly.

## 4 Discussion

### 4.1 Species Diversity Patterns and Influencing Factors

Understanding the spatial distribution patterns of species and their underlying mechanisms is crucial for predicting the impacts of environmental changes on biodiversity (Jetz et al. 2012). Our study on species identification and spatial diversity patterns within the Chengdu section of the GPNP provides valuable insights into the distribution and composition of mammal and gallinaceous bird species. Assessments of α‐diversity using the Shannon‐Wiener, Simpson, Chao1, and ACE indices indicated that the regions of DJYS and CZS exhibited relatively higher levels of species diversity, while PZS and QLS displayed lower values across most indices. In contrast, Pielou's evenness index did not reveal significant differences among these regions. The observed pattern is consistent with expectations of the “mid‐domain effect” (MDE), which proposes that overlapping species ranges may elevate diversity in central locations (Colwell et al. 2004; Currie and Kerr 2008; Dunn et al. 2007; Rangel and Diniz‐Filho 2005). Yet this central peak could equally reflect habitat heterogeneity, connectivity, protection history, or subtle human pressure. Consequently, the relatively high diversity in centrally located CZS, DJYS, and DYX may stem from the convergence of different species' ranges, potentially contributing to elevated species richness. Conversely, peripheral regions PZS and QLS experienced lower species richness apparently due to their more limited habitat overlap and greater distance from the core areas.

The β‐diversity analysis further elucidated the spatial variation in species composition, revealing that species turnover was the dominant factor contributing to faunal heterogeneity among regions, while nestedness had a relatively minor influence. This finding is consistent with studies in other local mountainous regions, which have shown that different regions within the GPNP support distinct species assemblages (Li et al. 2020, 2022; Wang et al. 2024). Furthermore, the highest pairwise turnover occurred between QLS and each of the PZS, CZS, and DYX regions, suggesting extensive species replacement and limited species overlap, possibly reflecting the land‐use and elevation gradient. The high species turnover in the Chengdu section of the GPNP indicates that spatial factors and local connectivity strongly shape species distributions, implying that conservation efforts should focus on maintaining ecological connectivity and integrity across the regions at the landscape scale to support the diverse species assemblages, rather than prioritizing species‐rich “source” regions based on nestedness patterns.

The spatial diversity patterns of species within ecosystems are shaped by an intricate array of abiotic and biotic factors, including climate variables, topography, habitat heterogeneity, and historical events. These factors also modulate species distribution dynamics and community composition through altitudinal gradients, vegetation structure, and microclimatic conditions (Hawkins et al. 2003; Heaney 2001). Giant panda populations in the Qinling and Qionglai Mountains adapt to their habitats through specific habitat selection and movement patterns, and climate, bamboo distribution, and landscape variables significantly influence the recent expansion of the giant panda population in the Qinling Mountains (Huang et al. 2020; Liu et al. 2015). At the regional scale, species turnover is likely driven by a complex interplay of ecological processes, including habitat fragmentation, environmental filtering, dispersal limitation, and species interactions (Arnan et al. 2015; Calderón‐Patrón et al. 2016; Legendre 2014). Environmental filtering restricts species distribution by selecting those adapted to specific conditions and plays a significant role in shaping species diversity patterns, while dispersal limitation determines species' ability to reach and colonize new habitats (Hawkins et al. 2003; Song et al. 2020). Therefore, the transformation of species distributions in the GPNP is hypothesized to result from both natural disturbances and direct or indirect human activities.

### 4.2 Conservation Recommendations and Future Directions

The high α‐diversity in DJYS and CZS underscores the necessity of conservation efforts to safeguard the rich species assemblages in these areas. Conversely, the lower species diversity in QLS, likely due to habitat fragmentation, highlights the need for targeted measures to improve habitat quality and connectivity. The high β‐diversity driven by species turnover indicates substantial compositional differences among districts, emphasizing the importance of enhanced cooperation among the various districts of Chengdu to boost biodiversity. Building on the GPNP's ongoing conservation efforts, maintaining or creating corridors would most likely support exchanges among distinct assemblages (Luo et al. 2024; Yang et al. 2025). However, habitat fragmentation, often a consequence of human activities such as deforestation and infrastructure development, can lead to isolated populations, reduced genetic diversity, and increased extinction risk for many species (Bai et al. 2023; Fahrig 1997; Liu et al. 2007, 2018; Wilson et al. 2016). Such fragmentation would disrupt inter‐regional connectivity, isolating distinct assemblages and eroding the β‐diversity documented herein. Future distribution prediction indicates that climate change may cause suitable giant panda habitats to shift eastward and then northwestward as temperatures rise, with the availability of suitable habitat potentially severely reduced (Ma et al. 2024). In response, strengthening habitat restoration and creating ecological corridors are essential to support species dispersal and gene flow, thereby promoting ecological resilience.

Over the past three decades, China's conservation efforts for the giant panda have achieved remarkable success, transforming this species from a symbol of endangered wildlife to an icon of conservation triumph (Rong et al. 2019). With the recovery of the wild population and the establishment of the GPNP, the focus of conservation has shifted from basic ecological research to more complex challenges, including landscape‐level connectivity restoration and multi‐species conservation planning, as maintaining biodiversity is essential for ecosystem health and resilience (Xu et al. 2022; Bai et al. 2023). These efforts necessitate the restoration of ecological functions of forests, mitigation of habitat fragmentation, and broader ecosystem conservation initiatives. For instance, habitat restoration experiments in the Daxiangling Mountains have demonstrated that thinning treatments in bamboo forests significantly increase bamboo sprouting rates and shoot growth (Yang et al. 2025). Meanwhile, existing studies have suggested that the GPNP should focus on measures such as restricting unreasonable infrastructure development, reducing human disturbance and grazing, implementing conservation education and ecological compensation, establishing habitat corridors, promoting natural habitat regeneration, and applying technical habitat restoration interventions (Luo et al. 2024).

This study offers several key conservation recommendations. First, our finding of high β‐diversity driven by species turnover among the five administrative regions underscores that enhancing regional cooperation and habitat connectivity is essential for maintaining biodiversity. Habitat restoration and the establishment of ecological corridors should be prioritized as conservation strategies, because current administrative boundaries do not align with ecological connectivity needs, and such measures can facilitate species dispersal and reduce the risk of local extinction. Second, the presence of distinct species assemblages across regions, including multiple rare and endangered species, underscores the need for conservation efforts to address both the giant panda and other sympatric species. The low detection rates of certain species in fragmented habitats further highlight the necessity of implementing long‐term monitoring programs to assess the effectiveness of conservation interventions and track changes in species diversity and distribution over time. Follow‐up monitoring of ecological restoration is also vital for evaluating the suitability of restored habitats for wildlife, as well as for tracking population growth, habitat use, and interspecific competition among these animals, thereby determining when and where management interventions are needed. Third, future research should continue to explore the underlying mechanisms driving species turnover and nestedness, as well as the potential impacts of climate change on these patterns.

These measures could help alleviate competition among sympatric species by providing more suitable habitats and improving connectivity, and enable the development and refinement of adaptive management strategies based on empirical data. This holistic approach will inform future management strategies for the effective conservation of both giant pandas and other sympatric species, ensuring the long‐term health and resilience of their shared ecosystems. While these recommendations offer a robust framework for conservation, it is worth noting that the field survey data span 2011–2014, and the results reflect patterns during this specific period that may not capture more recent land‐use or climate‐driven changes. Nevertheless, the findings provide essential insights into the spatial distribution patterns of species diversity in the Chengdu section of the GPNP, with significant implications for local biodiversity conservation, while also offering a reliable baseline for the upcoming 5th National Survey on Giant Pandas to validate current trends and assess future changes.

## 5 Conclusions

This study elucidated the spatial distribution patterns of species diversity in the Chengdu section of the Giant Panda National Park (GPNP), revealing distinct patterns of α‐ and β‐diversity among the five regions. The analysis indicated that the central areas of Chongzhou Shi (CZS), Dujiangyan Shi (DJYS), and Dayi Xian (DYX) exhibited higher species richness, while Pengzhou Shi (PZS) and Qionglai Shi (QLS) showed lower values, possibly reflecting the “mid‐domain effect” hypothesis. The β‐diversity results highlighted species turnover as the dominant component of faunal heterogeneity, while nestedness had a relatively minor influence. These findings highlight the importance of habitat connectivity and corridors, emphasizing the critical need for enhanced coordinated regional management to support biodiversity conservation. The results provide a scientific basis for targeted conservation strategies and long‐term monitoring, underscoring the importance of adaptive management approaches to ensure the ecological sustainability of the GPNP.

## Author Contributions

**Qianqian Wang:** conceptualization (lead), data curation (lead), formal analysis (lead), methodology (lead), visualization (lead), writing – original draft (lead), writing – review and editing (lead). **Chi Xu:** data curation (equal), investigation (equal), methodology (equal). **Yubo Gou:** formal analysis (equal). **Mingchun Zhang:** formal analysis (supporting). **Ke He:** formal analysis (supporting). **Qiujie Li:** formal analysis (supporting). **Jingjing Shuai:** methodology (supporting). **Chun Yin:** methodology (supporting). **Zhaowen Wang:** writing – review and editing (supporting). **Zhisong Yang:** formal analysis (supporting), methodology (supporting), writing – review and editing (supporting). **Biao Yang:** conceptualization (equal), data curation (equal), funding acquisition (lead), project administration (lead), resources (supporting), supervision (supporting), writing – review and editing (supporting).

## Funding

This study was partially supported by the National Natural Science Foundation of China (32470535) and Startup Fund for Introduced Talents of China Conservation and Research Center for the Giant Panda (CCRCGPRC202503).

## Conflicts of Interest

The authors declare no conflicts of interest.

## Supporting information


**Data S1:** ece373180‐sup‐0001‐Supinfo.docx.

## Data Availability

The original data of the trace points for giant pandas and their sympatric species are available from Zenodo: https://doi.org/10.5281/zenodo.17168371.
