# Supplementary material for: Species Identification and Spatial Diversity Patterns of the Giant Panda National Park (GPNP) in Chengdu, Sichuan, China
Source: Ecol Evol. 2026 Feb 27;16(3):e73180. doi: 10.1002/ece3.73180 (PMC12949331; doi:10.1002/ece3.73180)
Supplement: Supplementary file 1 — Data S1: ece373180‐sup‐0001‐Supinfo.docx. [file ECE3-16-e73180-s001.docx]

**TABLE S1** List of species monitored in the study area of the GPNP in Chengdu, Sichuan, China.

| Order, Family, and Species names | IUCN Red List Status | China’s National Protected Category | Species trace points recording frequency | | | | | |
| --- | --- | --- | --- | --- | --- | --- | --- | --- |
|  |  |  | PZS | DJYS | CZS | DYX | QLS | Total |
| **Primates** |  |  |  |  |  |  |  |  |
| **Cercopithecidae** |  |  |  |  |  |  |  |  |
| Golden snub-nosed monkey *Rhinopithecus roxellana* | EN | Ⅰ | 0 | 13 | 1 | 1 | 0 | 15 |
| Tibetan macaque *Macaca thibetana* | NT | Ⅱ | 0 | 13 | 12 | 1 | 0 | 26 |
| Rhesus monkey *Macaca mulatta* | LC | Ⅱ | 0 | 1 | 1 | 2 | 0 | 4 |
| **Artiodactyla** |  |  |  |  |  |  |  |  |
| **Cervidae** |  |  |  |  |  |  |  |  |
| Tufted deer *Elaphodus cephalophus* | NT | Ⅱ | 1 | 29 | 17 | 17 | 1 | 65 |
| Reeves’ muntjac *Muntiacus reevesi* | LC | / | 0 | 1 | 0 | 0 | 0 | 1 |
| Sambar *Rusa unicolor* | VU | Ⅱ | 0 | 0 | 11 | 4 | 0 | 15 |
| Wapiti *Cervus elaphus* | LC | Ⅱ | 0 | 0 | 0 | 1 | 0 | 1 |
| **Bovidae** |  |  |  |  |  |  |  |  |
| Chinese Serow *Capricornis milneedwardsii* | VU | Ⅱ | 2 | 11 | 2 | 6 | 0 | 21 |
| Blue sheep *Pseudois nayaur* | LC | Ⅱ | 1 | 5 | 2 | 14 | 0 | 22 |
| Takin *Budorcas tibetana* | VU | Ⅰ | 5 | 81 | 69 | 55 | 1 | 211 |
| Chinese goral *Naemorhedus griseus* | VU | Ⅱ | 6 | 44 | 17 | 22 | 0 | 89 |
| **Moschidae** |  |  |  |  |  |  |  |  |
| [Forest musk deer](https://baike.so.com/doc/6934529-7156881.html) *Moschus berezovskii* | EN | Ⅰ | 4 | 6 | 6 | 6 | 0 | 22 |
| **Suidae** |  |  |  |  |  |  |  |  |
| Wild boar *Sus scrofa* | LC | / | 4 | 38 | 20 | 25 | 2 | 89 |
| **Carnivora** |  |  |  |  |  |  |  |  |
| **Ailuropodidae** |  |  |  |  |  |  |  |  |
| Giant panda *Ailuropoda melanoleuca* | VU | Ⅰ | 22 | 44 | 77 | 70 | 0 | 213 |
| **Ursidae** |  |  |  |  |  |  |  |  |
| Asiatic black bear *Ursus thibetanus* | VU | Ⅱ | 2 | 7 | 5 | 3 | 0 | 17 |
| **Felidae** |  |  |  |  |  |  |  |  |
| Mainland leopard cat *Prionailurus bengalensis* | LC | Ⅱ | 5 | 28 | 13 | 7 | 0 | 53 |
| **Ailuridae** |  |  |  |  |  |  |  |  |
| Red panda *Ailurus styani* | EN | Ⅱ | 0 | 1 | 31 | 36 | 0 | 68 |
| **Viverridae** |  |  |  |  |  |  |  |  |
| Masked palm civet *Paguma larvata* | LC | / | 0 | 1 | 0 | 0 | 1 | 2 |
| **Mustelidae** |  |  |  |  |  |  |  |  |
| Hog badger *Arctonyx collaris* | VU | / | 1 | 0 | 1 | 1 | 0 | 3 |
| Asian badger *Meles meles* | LC | / | 0 | 1 | 0 | 0 | 0 | 1 |
| Small-toothed ferret badger *Melogale moschata* | LC | / | 0 | 0 | 0 | 0 | 1 | 1 |
| **Mustelidae** |  |  |  |  |  |  |  |  |
| Siberian weasel *Mustela sibirica* | LC | / | 1 | 0 | 1 | 0 | 0 | 2 |
| **Rodentia** |  |  |  |  |  |  |  |  |
| **Sciuridae** |  |  |  |  |  |  |  |  |
| Pallas’s squirrel *Callosciurus erythraeus* | LC | / | 0 | 0 | 0 | 1 | 0 | 1 |
| **Hystricidae** |  |  |  |  |  |  |  |  |
| Chinese porcupine *Hystrix hodgsoni* | LC | / | 0 | 2 | 0 | 1 | 0 | 3 |
| **Galliformes** |  |  |  |  |  |  |  |  |
| **Phasianidae** |  |  |  |  |  |  |  |  |
| Golden pheasant *Chrysolophus pictus* | LC | Ⅱ | 1 | 3 | 3 | 1 | 0 | 8 |
| Blood pheasant *Ithaginis cruentus* | LC | Ⅱ | 0 | 0 | 0 | 3 | 0 | 3 |
| Chinese bamboo partridge *Bambusicola thoracicus* | LC | / | 0 | 1 | 0 | 0 | 1 | 2 |
| Chinese monal pheasant *Lophophorus lhuysii* | VU | Ⅰ | 0 | 0 | 0 | 1 | 0 | 1 |

*Note*: IUCN Red List: EN, Endangered; VU, Vulnerable; NT, Near Threatened; LC, Least Concern. Categories Ⅰ and Ⅱ correspond to first - class and second - class of China’s National Key Protected Wildlife List, respectively.

**TABLE S2** The results of α-diversity indices analysis of the five regions within the GPNP in Chengdu.

| Five regions | Shannon-Wiener index | Simpson index | Pielou index | $S_{chao1}$ | S_ACE_ |
| --- | --- | --- | --- | --- | --- |
| PZS | 2.01063 | 0.78954 | 0.78389 | 16.33333 | 16.98438 |
| DJYS | 2.31937 | 0.87062 | 0.77423 | 27.50000 | 28.22630 |
| CZS | 2.19331 | 0.84261 | 0.75883 | 20.00000 | 22.09524 |
| DYX | 2.28576 | 0.85790 | 0.73948 | 36.00000 | 32.21042 |
| QLS | 1.74787 | 0.81633 | 0.97550 | 11.00000 | 21.00000 |

**TABLE S3** The β-diversity decomposition results of the five regions within the GPNP in Chengdu by using Jaccard and Sørensen dissimilarity.

| Methods | Turnover | Nestedness | Total dissimilarity |
| --- | --- | --- | --- |
| Jaccard dissimilarity | 0.45161 (64.35%) | 0.25014 (35.65%) | 0.70175 |
| Sørensen dissimilarity | 0.29167 (53.96%) | 0.24887 (46.04%) | 0.54054 |

*Note*: The percentage in parentheses indicates the proportion that this component accounts for the total dissimilarity.

**TABLE S4** Pairwise Jaccard dissimilarities of the five regions within the GPNP in Chengdu.

| Jaccard dissimilarity | PZS | DJYS | CZS | DYX | QLS |
| --- | --- | --- | --- | --- | --- |
| PZS |  | 0.2666667 | 0.0000000 | 0.1428571 | 0.6666667 |
| DJYS | 0.5000000 |  | 0.2857143 | 0.3333333 | 0.2857143 |
| CZS | 0.2777778 | 0.3478261 |  | 0.1052632 | 0.6666667 |
| DYX | 0.4782609 | 0.3846154 | 0.2608696 |  | 0.6666667 |
| QLS | 0.8125000 | 0.7619048 | 0.8571429 | 0.8800000 |  |

*Note:* Values in the lower-left triangle give the pairwise β-diversity dissimilarity, whereas those in the upper-right triangle represent the turnover component. The same notation applies to the table below.

**TABLE S5** Pairwise Sørensen dissimilarities of the five regions within the GPNP in Chengdu.

| Sørensen dissimilarity | PZS | DJYS | CZS | DYX | QLS |
| --- | --- | --- | --- | --- | --- |
| PZS |  | 0.15384615 | 0.0000000 | 0.07692308 | 0.50000000 |
| DJYS | 0.3333333 |  | 0.16666667 | 0.20000000 | 0.16666667 |
| CZS | 0.1612903 | 0.2105263 |  | 0.05555556 | 0.50000000 |
| DYX | 0.3142857 | 0.2380952 | 0.1500000 |  | 0.50000000 |
| QLS | 0.6842105 | 0.6153846 | 0.7500000 | 0.7857143 |  |
